# Impact of Different Economic Factors on Biological Invasions on the Global Scale

**DOI:** 10.1371/journal.pone.0018797

**Published:** 2011-04-13

**Authors:** Wen Lin, Xinyue Cheng, Rumei Xu

**Affiliations:** 1 Zhangzhou Entry-Exit Inspection and Quarantine Bureau, Zhangzhou, Fujian, China; 2 State Key Laboratory of Earth Surface Processes and Resource Ecology, Beijing Normal University, Beijing, China; Tel Aviv University, Israel

## Abstract

Social-economic factors are considered as the key to understand processes contributing to biological invasions. However, there has been few quantified, statistical evidence on the relationship between economic development and biological invasion on a worldwide scale. Herein, using principal factor analysis, we investigated the relationship between biological invasion and economic development together with biodiversity for 91 economies throughout the world. Our result indicates that the prevalence of invasive species in the economies can be well predicted by economic factors (R^2^ = 0.733). The impact of economic factors on the occurrence of invasive species for low, lower-middle, upper-middle and high income economies are 0%, 34.3%, 46.3% and 80.8% respectively. Greenhouse gas emissions (CO_2_, Nitrous oxide, Methane and Other greenhouse gases) and also biodiversity have positive relationships with the global occurrence of invasive species in the economies on the global scale. The major social-economic factors that are correlated to biological invasions are different for various economies, and therefore the strategies for biological invasion prevention and control should be different.

## Introduction

Invasions by non-indigenous species are a growing global problem [1]. In today's world, almost all countries suffer similar problems from the effects of invasive species, while they are also exporters of invaders to other countries. Alien invasive plants, animals and pathogens caused serious environmental and economic damages and have altered ecosystems throughout the world. Biological invasions are considered as the second most important threat to biodiversity [2], [3]. The intensive global trade and transportation has been blamed to be the major cause of biological invasions [4]. Social-economic factors are considered as the key to understand processes contributing to biological invasions [5]–[8]. “The causes of the problem of invasive alien species are primarily economic and, as such, require economic solutions” [9].

Lacking from our current theories of human-induced species invasions is the explicit integration of ecological and economic causal pathways [6]. So far, there are few quantified and statistical evidence on the relationship between economic development and biological invasion on the worldwide scale. We had proven that economic developments had accelerated biological invasion in China, and the prevalence of invasive species can be well predicted by the economic development on the provincial scale [10]. Is this rule applicable on the global scale? There is a strong geographical bias in the regions of the globe where research on biological invasions is conducted [11]. These differences in data quality and availability create a challenge in forming global strategies to deal with invasions [8]. If the driving economic factors are not the same for biological invasions in different economies, the strategies for the prevention and control of invasive species should have what differences? These are all important questions for us to explore for a better understanding, prediction and management of invasive species.

## Results

Our results indicate that high-income economies have more invasive species. The top 5 economies which have the highest numbers of invasive species are all high-income economies (Table 1).

**Table 1:** Top 5 economies ranked by the number of invasive species.

Ranked by Number of Invasive Species	Country's Name	Number of Invasive Species	Country's Type	Ranked by GDP
1	United States	447	H	1
2	Australia	247	H	15
3	Canada	137	H	8
4	France	100	H	5
5	United Kingdom	89	H	4

H: High-income Economies.

Based on the invasive species data collected from Global Invasive Species Database (GISD), and economic and biodiversity data collected from The World Development Indicators (WDI), The World Factbook and Species 2000, we found that 27 variables have significant associations with the number of invasive species for all economies throughout the world (p<0.05). Through principal factor analysis, four principal components were selected; the contribution rate is 59.19%, 11.65%, 10.75% and 9.75% of the total variance respectively (Table 2). The 1st component consists mainly of economic variables in which GDP, imports and services have the highest load (0.971, 0.961 and 0.960, respectively). The 2nd component includes human population and agriculture value. The 3rd principal component reflects biodiversity. The 4th component includes forest area, land area and waterway.

**Table 2:** Result of the principal factor analysis for 91 economies.

		Factor loadings‡			
Variables†		1	2	3	4
Gross domestic product		0.971	−0.113	0.023	−0.133
Imports of goods and services		0.961	−0.048	−0.052	−0.162
Services, etc., value added		0.960	−0.167	0.013	−0.138
Industry, value added		0.956	0.023	0.042	−0.144
Energy use		0.945	0.244	0.065	0.145
Railway		0.922	0.050	0.072	0.309
International tourism, receipts		0.917	−0.116	−0.038	−0.130
International migrant stock, total		0.917	−0.202	−0.079	0.172
CO_2_ emissions		0.908	0.337	0.068	0.140
Exports of goods and services		0.898	0.039	−0.075	−0.169
Roadway		0.889	0.081	0.218	0.084
International tourism, expenditures		0.885	−0.091	−0.103	−0.186
Other greenhouse gas emissions, HFC, PFC and SF_6_		0.874	0.331	−0.022	0.116
Airports		0.856	−0.265	0.194	0.186
Energy production		0.803	0.319	0.091	0.430
Net migration		0.742	−0.482	−0.260	0.122
Nitrous oxide emissions		0.705	0.526	0.363	0.150
Methane emissions		0.681	0.542	0.273	0.350
Agricultural land		0.569	0.380	0.413	0.396
Population, total		0.392	0.852	0.223	0.091
Agriculture, value added		0.677	0.691	0.211	0.017
Plant species (higher); total known		0.204	0.132	0.872	0.185
GEF benefits index for biodiversity		0.465	0.080	0.817	0.213
Species, total known		0.410	0.341	0.795	0.074
Forest area		0.410	−0.021	0.245	0.828
Land area		0.508	0.147	0.253	0.772
Waterway		0.461	0.512	0.204	0.610
Rotated sums of squared loadings§	Eigenvalues	15.981	3.145	2.902	2.631
	% of variance	59.190	11.649	10.748	9.746
	Cumulative %	59.190	70.839	81.587	91.333

† Refer to Table S2 for details and units.

‡ Extraction method was Principal component analysis.

§ Rotation method was Quartimax with Kaizer Normalization.

A multiple regression model was established between the number of invasive species and the factor scores of each component. The first three principal components were selected and they accounted for 83.2% of the total variance in the number of invasive species, indicating a significant association between biological invasion and those factors (F_3, 87_ = 143.906, p<0.001). Economic factors proved most important, influencing the occurrence of invasive species (R^2^ = 0.733). Biodiversity, population and agriculture constitute the next two most important components (R^2^ = 0.064 and 0.035, respectively) (Table 3).

**Table 3:** Stepwise regression between number of invasive species and factor scores of the principal components for 91 economies.

Variable entered by stepwise order	Regression		Analysis of variance (ANOVA)		
	Coefficients	R^2^ †	d. f.	F	Significance
Constant	37.791				
Factor 1‡	47.152	0.733	1, 89	243.815	<0.001
Factor 3‡	14.012	0.797	2, 88	173.040	<0.001
Factor 2‡	−10.307	0.832	3, 87	143.906	<0.001

† Step by step cumulative R^2^.

‡ Factor Score 1, Factor Score 3 and Factor Score 2 correspond to Principal components 1, 3 and 2 in Table 2.

## Discussion

**Figure 1:**
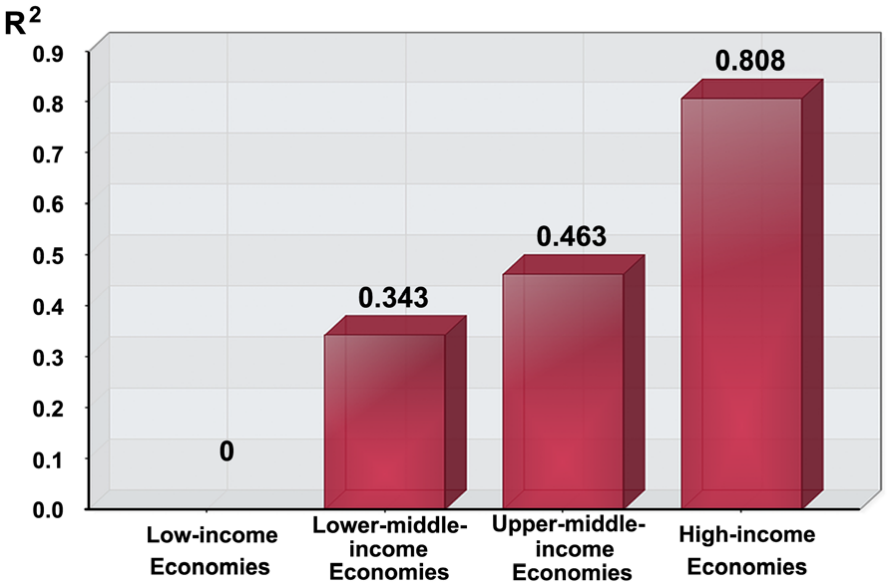
The impact of economic components (R^2^) on the number of invasive species for different income-groups.

**Figure 2:**
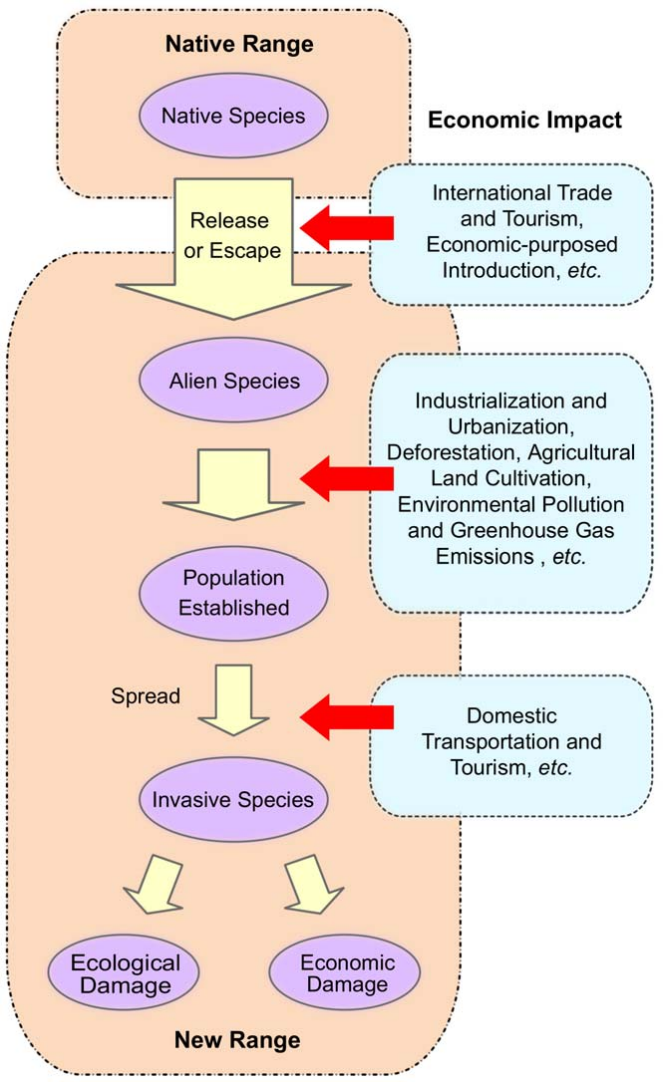
Economic activities promote biological invasions acting on the different transfer stages of biological invasions.

Economic development has heavier impact on the distribution of invasive species in the economies with higher levels of economic development (Figure 1) (Table 4, 5, 6, 7, Table S3, S4, S5, S6). In low-income economies, there is no significant relationship between economic development and the number of invasive species, but mainly determined by international population flow (R^2^ = 0.752, F_1, 8_ = 24.214, p<0.002). In low-income, lower-middle-income, upper-middle-income and high-income economies, economic impacts are increasing (R^2^ = 0, 0.343, 0.463 and 0.808, respectively). Biological invasion is a complex chain process [1], [12]. Accompanying economic developments, economic activities promote the occurrence and success for the invader in each step of the invasion process (Figure 2). Economic and other human factors enhance international trade, travel and economic-purposed introduction that transport alien species to new areas. They accelerate industrialization and urbanization that are responsible for disturbances of nature habitats that allow invasive species to establish, intensifies the loss of resistance from the local communities to invasions. They are also influence the domestic transportation and travel, and thus enhance the spread of invasive species. Thus, when the rate of success increases in each step of the chain process, the total probability of a successful invasion will be highly promoted according to the tens rule of Williamson [12].

**Table 4:** Result of the principal factor analysis for high-income economies.

		Factor loadings‡		
Variables†		1	2	3
Energy use		0.992	−0.020	0.033
CO_2_ emissions		0.988	−0.006	0.046
Services, etc., value added		0.981	−0.033	−0.120
International migrant stock, total		0.981	−0.013	0.129
Railway		0.981	0.107	0.138
Gross domestic product		0.980	−0.035	−0.143
Roadway		0.979	0.092	0.127
Nitrous oxide emissions		0.971	0.125	0.140
Population, total		0.971	−0.038	0–.211
Methane emissions		0.969	0.083	0.202
Net migration		0.957	−0.012	0.163
Imports of goods and services		0.956	−0.138	−0.156
Energy production		0.952	0.088	0.246
Waterway		0.950	−0.009	0.149
Airports		0.949	−0.015	0.268
Industry, value added		0.943	−0.016	−0.266
Agriculture, value added		0.927	0.050	−0.273
International tourism, receipts		0.924	−0.055	−0.002
Other greenhouse gas emissions, HFC, PFC and SF_6_		0.920	−0.039	−0.342
Exports of goods and services		0.862	−0.166	−0.322
International tourism, expenditures		0.848	−0.144	−0.344
Plant species (higher); total known		0.747	0.562	0.039
Forest area		0.692	0.432	0.362
Land area		0.646	0.575	0.341
Species, total known		0.555	0.795	−0.225
GEF benefits index for biodiversity		0.690	0.711	−0.032
Agricultural land		0.674	0.679	0.163
Rotated sums of squared loadings§	Eigenvalues	21.733	2.562	1.212
	% of variance	80.491	9.489	4.487
	Cumulative %	80.491	89.980	94.468

† Refer to Table S2 for details and units.

‡ Extraction method was Principal component analysis.

§ Rotation method was Quartimax with Kaizer Normalization.

**Table 5:** Result of the principal factor analysis for upper-middle-income economies.

		Factor loadings‡	
Variables†		1	2
Gross domestic product		0.989	−0.012
Industry, value added		0.982	−0.091
Services, etc., value added		0.980	−0.005
Population, total		0.942	0.137
Agriculture, value added		0.920	0.037
Exports of goods and services		0.883	−0.388
Imports of goods and services		0.875	−0.377
Airports		0.865	0.358
GEF benefits index for biodiversity		0.827	0.489
International tourism, expenditures		0.781	−0.382
Nitrous oxide emissions of CO_2_		0.760	0.525
Species, total known		0.720	0.596
International tourism, receipts		0.641	−0.589
Plant species (higher); total known		0.633	0.637
Rotated sums of squared loadings§	Eigenvalues	10.131	2.218
	% of variance	72.366	15.845
	Cumulative %	72.366	88.212

† Refer to Table S2 for details and units.

‡ Extraction method was Principal component analysis.

§ Rotation method was Quartimax with Kaizer Normalization.

**Table 6:** Result of the principal factor analysis for lower-middle-income economies.

		Factor loadings‡		
Variables†		1	2	3
Gross domestic product		0.997	0.031	−0.032
Energy use		0.995	0.036	−0.055
Services, etc., value added		0.993	0.085	−0.029
CO_2_ emissions		0.993	−0.023	−0.079
Industry, value added		0.987	−0.113	−0.033
Agriculture, value added		0.984	0.153	−0.029
Imports of goods and services		0.984	−0.078	−0.035
Energy production		0.983	−0.037	−0.004
Nitrous oxide emissions		0.983	0.131	−0.040
International tourism, expenditures		0.982	−0.043	0.026
Exports of goods and services		0.980	−0.117	−0.024
Waterway		0.969	−0.188	0.032
Land area		0.964	−0.101	0.002
Other greenhouse gas emissions, HFC, PFC and SF_6_		0.957	−0.193	−0.127
Agricultural land		0.951	−0.082	−0.097
Methane emissions		0.951	0.273	0.037
Species, total known		0.943	0.093	0.205
Population, total		0.926	0.344	−0.002
International tourism, receipts		0.896	−0.137	−0.022
Railway		0.889	0.359	−0.088
Forest area		0.866	−0.141	0.306
Plant species (higher); total known		0.723	−0.008	0.643
Net migration		−0.721	−0.426	−0.234
Population density		0.273	0.830	0.018
Roadway		0.661	0.670	0.034
GEF benefits index for biodiversity		0.648	0.140	0.727
Rotated sums of squared loadings§	Eigenvalues	21.382	1.884	1.187
	% of variance	82.239	7.244	4.565
	Cumulative %	82.239	89.483	94.048

† Refer to Table S2 for details and units.

‡ Extraction method was Principal component analysis.

§ Rotation method was Quartimax with Kaizer Normalization.

**Table 7:** Result of the principal factor analysis for low-income economies.

		Factor loadings‡
Variables†		1
International migrant stock, total		0.936
International tourism, expenditures		0.819
Energy use		0.749
International tourism, receipts		0.645
Sums of squared loadings	Eigenvalues	2.523
	% of variance	63.083
	Cumulative %	63.083

† Refer to Table S2 for details and units.

‡ Extraction method was Principal component analysis.

All of the 4 greenhouse gases emission variables (CO_2_, Nitrous oxide, Methane and Other greenhouse gas emissions) have positive relationships (p<0.001) with the number of invasive species for all economies throughout the world. Especially, CO_2_ emission has a rather high load in the economic component for high-income and lower-middle-income economies (ranked 2^nd^ and 4^th^ respectively) (Table 4 and Table 6). Recent studies have indicated that increase in atmospheric CO_2_ concentration may facilitate biological invasions [13]–[16]. The response of invasive species and native species are different to elevated CO_2_ [17] and invasive species showed a greater increase in energy-use efficiency under elevated CO_2_ [18]. Increased soil N availability may often facilitate plant invasions [13], [19]–[22].

Also, our results indicated that biodiversity has a strong positive relationship with the number of invasive species on the global scale (p<0.001). The relationship between biodiversity and biological invasions has been in debate for many decades since the publication of Elton [23]. The relationships are often negative on a small scale, but positive on a large scale [24]–[27]. At community-wide scales, the effects of ecological factors spatially co-varying with diversity, make the most diverse communities most likely to be invaded [28]. The changes in the number of available resources across communities can cause invasion success to become positively correlated with native species diversity at larger scales [29]. Our result presented evidence that biodiversity and biological invasion is positively related on the global scale.

The major social-economic factors that are correlated to biological invasions are different for various economies, and therefore the strategies for biological invasion prevention and control should be different:

### 1. High-income Economies

The 1^st^ component consists of economic factors (contribution rate  = 80.49% of the total variance). Energy use, CO_2_ emissions, services, international migrant stock and railway have the highest load (0.992, 0.988, 0.981, 0.981 and 0.981, respectively) (Table 4). The 1^st^ component accounted for 80.8% of the total variance in the number of invasive species (F_3, 24_ = 263.532, p<0.001) (Table S3). High-income economies, with just 15 percent of world population, use almost half of global energy [30]. Therefore, for these economies, reduce energy use and greenhouse gas emissions are important actions for obtaining a greener GDP, but often being overlooked by the public for reducing the risk of biological invasions.

### 2. Low-income Economies

The only component consists of international migrant stock, international tourism expenditures, energy use and international tourism receipts (contribution rate  = 63.08% of the total variance). They have the load of 0.936, 0.819, 0.749 and 0.645, respectively (Table 7). The component accounted for 75.2% of the total variance in number of invasive species (F_1, 8_ = 24.214, p<0.002) (Table S6). For these economies, strengthen inspection at important ports to prevent the introduction of alien species is the most important action to prevent biological invasions.

### 3. Middle-income Economies

These two categories of economies have more similarities, though economic factors have more impact for the upper-middle-income economies. For the lower-middle-income economies, the 1^st^ component consists of economic factors (contribution rate  = 82.24% of the total variance). GDP, energy use, services, CO_2_ emissions have the highest load (0.997, 0.995, 0.993 and 0.993 respectively) (Table 6). The 1^st^ component accounted for 34.3% of the total variance in number of invasive species. The 2^nd^ component (population and roadway) and the 3^rd^ component (biodiversity) accounted for 13.9% and 29.2% (F_3, 25_ = 28.597, p<0.001) (Table S5). For the upper-middle-income economies, the 1^st^ component consists of economic factors (contribution rate  = 72.37% of the total variance). GDP, industry, services have the highest load (0.989, 0.982 and 0.980 respectively) (Table 5). The 1^st^ component accounted for 46.3% of the total variance in number of invasive species (F_1, 22_ = 19.002, p<0.001) (Table S4).

As could be seen, these economies are in a more complex situation. The factors are more diverse. For these economies, the strategies suggested for developed economies are not enough, and those for the low-income economies are too simple. Fortunately, we have investigated a case study using China as a model [10]. We demonstrated that the increase in biological invasion was coincident with the rapid economic development that had occurred in China over the past three decades. Economic impact (R^2^ = 0.379) is similar, if not more important than climatic factors (R^2^ = 0.345). We unexpectedly found that residential construction had the strongest positive effect on the occurrence of invasive species. However, it is not hard to explain. From 1995 to 2004, residential construction in China increased at the average rate of 15.3% per year [31]. It is reported that nearly half of the world's buildings under construction are located in China [32]. Such rapid increase in residential construction and expansion of small towns facilitates timber transportation, urbanization, the degradation and fragmentation of habitats, and therefore, the actions needed (e.g., ecological city construction) to block out these pathways can also be clarified and be taken to reduce invasions. The implement of ecological city planning, sustainable industry and the augmentation of inter-province inspection and quarantine should also be further stressed for restricting the spread of invasive species in China. The China investigation can be used here as a sample. Various economies have different ways of economic developments, and maybe this is the reason why the factors influencing biological invasions are so diverse. We suggest, for each different economy, investigations are required to pin point their specific economic factors and their specific impact on biological invasion, and thus, to obtain a better strategy for management and control.

In summary, the super-complexity of the biological processes involved, interacting with the extreme stochastic of human activities makes the understanding and prediction of biological invasions a very difficult task [33]. The actual ecological-economical pathways and mechanisms underlying the interactions between different economic factors and biological invasions for various economies is urgently in need to be stressed for further investigation, to achieve a better understanding, prevention and control of invasive species. Therefore, the task of investigating and prevention of invasive species is not only the task for biologists.

## Materials and Methods

### Data collection

We collected the number of invasive species from Global Invasive Species Database (GISD). Economic and biodiversity data was collected from 2000 to 2006 from The World Development Indicators (WDI), The World Factbook and Species 2000. Because of the lacking of data, only 91 economies were selected, which were divided into 4 groups (Table S1) according to 2008 GNI per capita, calculated using the World Bank Atlas method. Based on linear regressions between economic variables and the number of invasive species in each economy, 28 variables were selected (Table S2). The mean values of these variables were used for data analysis.

### Data analysis

Principal factor analysis was carried out on these economic and diversity variables. The number of principal components we selected is based on Kaiser criteria. After analysis using Quartimax with Kaiser normalization rotation, we further removed those variables with absolute load<0.5. The remaining variables were subject to final principal factor analysis and a factor score for each economy was given accordingly. A multiple regression model was established between the number of invasive species and the factor scores of each economies, through stepwise selection method with p = 0.10 entering and p = 0.05 removing criteria.

## Supporting Information

Table S1 The list of 4 income-groups of 91 economies.
(DOC)

Table S2 List of variables used for analysis.
(DOC)

Table S3 Stepwise regression between number of invasive species and factor scores of the principal components for high-income economies.
(DOC)

Table S4 Stepwise regression between number of invasive species and factor scores of the principal components for upper-middle-income economies.
(DOC)

Table S5 Stepwise regression between number of invasive species and factor scores of the principal components for lower-middle-income economies.
(DOC)

Table S6 Stepwise regression between number of invasive species and factor scores of the principal components for low-income economies.
(DOC)
