# Supplementary material for: Impact of Different Economic Factors on Biological Invasions on the Global Scale
Source: PLoS One. 2011 Apr 13;6(4):e18797. doi: 10.1371/journal.pone.0018797 (PMC3076446; doi:10.1371/journal.pone.0018797)
Supplement: Table S1 — The list of 4 income-groups of 91 economies. (DOC) [file pone.0018797.s001.doc]

Table S1: The list of 4 income-groups of 91 economies

| **Low-income Economies (10, GNI per capita: $975 or less):** | |
| --- | --- |
| Bangladesh | Mozambique |
| Benin | Senegal |
| Ethiopia | Togo |
| Ghana | Yemen, Rep. of |
| Kyrgyz Republic | Zambia |

| **Lower-middle-income Economies (29, GNI per capita: $976 - $3,855):** | |
| --- | --- |
| Albania | Moldova |
| Angola | Mongolia |
| Azerbaijan | Morocco |
| Bolivia | Nicaragua |
| Cameroon | Nigeria |
| China | Pakistan |
| Ecuador | Paraguay |
| Egypt, Arab Rep. | Philippines |
| Georgia | Sri Lanka |
| Guatemala | Sudan |
| Honduras | Syrian Arab Republic |
| India | Thailand |
| Indonesia | Tunisia |
| Iran, Islamic Rep. of | Ukraine |
| Jordan |  |

| **Upper-middle-income Economies (24, GNI per capita: $3,856 - $11,905):** | |
| --- | --- |
| Algeria | Latvia |
| Argentina | Lebanon |
| Botswana | Malaysia |
| Brazil | Mexico |
| Bulgaria | Namibia |
| Chile | Panama |
| Colombia | Peru |
| Costa Rica | Poland |
| Dominican Republic | Russian Federation |
| Gabon | Turkey |
| Jamaica | Uruguay |
| Kazakhstan | Venezuela, R.B. de |

| **High-income Economies (28, GNI per capita: $11,906 or more):** | |
| --- | --- |
| Australia | Ireland |
| Austria | Italy |
| Belgium | Japan |
| Brunei Darussalam | Korea, Rep. of |
| Canada | Luxembourg |
| Croatia | Netherlands |
| Czech Republic | Norway |
| Denmark | Oman |
| Estonia | Portugal |
| Finland | Singapore |
| France | Slovak Republic |
| Germany | Spain |
| Greece | United Kingdom |
| Iceland | United States |
