# Supplementary material for: Impact of Different Economic Factors on Biological Invasions on the Global Scale
Source: PLoS One. 2011 Apr 13;6(4):e18797. doi: 10.1371/journal.pone.0018797 (PMC3076446; doi:10.1371/journal.pone.0018797)
Supplement: Table S2 — List of variables used for analysis. (DOC) [file pone.0018797.s002.doc]

**Table S2: List of variables used for analysis**

| **Variables** | **Units** |
| --- | --- |
| Gross Domestic Product | current U.S. dollars |
| Agriculture, value added | current U.S. dollars |
| Industry, value added | current U.S. dollars |
| Services, etc., value added | current U.S. dollars |
| Exports of goods and services | current U.S. dollars |
| Imports of goods and services | current U.S. dollars |
| Population, total |  |
| Population density | people per sq. km |
| International migrant stock, total |  |
| Net migration |  |
| Agricultural land | sq. km |
| Forest area | sq. km |
| Land area | sq. km |
| GEF benefits index for biodiversity | From 0 to100 |
| Species, total known |  |
| Plant species (higher); total known |  |
| International tourism, expenditures | current U.S. dollars |
| International tourism, receipts | current U.S. dollars |
| Railway | km |
| Roadway | km |
| Waterway | km |
| Airports |  |
| CO_2_ emissions | kt |
| Methane emissions | kt of CO_2_ equivalent |
| Nitrous oxide emissions | thousand metric tons of CO_2_ equivalent |
| Other greenhouse gas emissions, HFC, PFC and SF_6_ | thousand metric tons of CO_2_ equivalent |
| Energy production | kt of oil equivalent |
| Energy use | kt of oil equivalent |
