# Supplementary material for: Impact of Different Economic Factors on Biological Invasions on the Global Scale
Source: PLoS One. 2011 Apr 13;6(4):e18797. doi: 10.1371/journal.pone.0018797 (PMC3076446; doi:10.1371/journal.pone.0018797)
Supplement: Table S3 — Stepwise regression between number of invasive species and factor scores of the principal components for high-income economies. (DOC) [file pone.0018797.s003.doc]

Table S3: Stepwise regression between number of invasive species and factor scores of the principal components for high-income economies

| Variable entered by stepwise order | Regression |  | Analysis of variance (ANOVA) | | |
| --- | --- | --- | --- | --- | --- |
|  | Coefficients | R^2†^ | d. f. | F | Significance |
| Constant | 64.786 |  |  |  |  |
| Factor 1^‡^ | 81.135 | 0.808 | 1, 26 | 109.362 | <0.001 |
| Factor 2^‡^ | 35.358 | 0.961 | 2, 25 | 310.993 | <0.001 |
| Factor 3^‡^ | 8.648 | 0.971 | 3, 24 | 263.532 | <0.001 |
| ^†^ Step by step cumulative R^2^. | | | | | |
| ^‡^ Factor Score 1, Factor Score 2 and Factor Score 3 correspond to Principal components 1, 2 and 3 in Table 4. | | | | | |
