# Supplementary material for: Impact of Different Economic Factors on Biological Invasions on the Global Scale
Source: PLoS One. 2011 Apr 13;6(4):e18797. doi: 10.1371/journal.pone.0018797 (PMC3076446; doi:10.1371/journal.pone.0018797)
Supplement: Table S4 — Stepwise regression between number of invasive species and factor scores of the principal components for upper-middle-income economies. (DOC) [file pone.0018797.s004.doc]

Table S4: Stepwise regression between number of invasive species and factor scores of the principal components for upper-middle-income economies

| Variable entered by stepwise order | Regression |  | Analysis of variance (ANOVA) | | |
| --- | --- | --- | --- | --- | --- |
|  | Coefficients | R^2†^ | d. f. | F | Significance |
| Constant | 32.083 |  |  |  |  |
| Factor 1^‡^ | 13.028 | 0.463 | 1, 22 | 19.002 | <0.001 |
| ^†^ Step by step cumulative R^2^. | | | | | |
| ^‡^ Factor Score 1 correspond to Principal components 1 in Table 5. | | | | | |
