# Supplementary material for: Impact of Different Economic Factors on Biological Invasions on the Global Scale
Source: PLoS One. 2011 Apr 13;6(4):e18797. doi: 10.1371/journal.pone.0018797 (PMC3076446; doi:10.1371/journal.pone.0018797)
Supplement: Table S5 — Stepwise regression between number of invasive species and factor scores of the principal components for lower-middle-income economies. (DOC) [file pone.0018797.s005.doc]

Table S5: Stepwise regression between number of invasive species and factor scores of the principal components for lower-middle-income economies

| Variable entered by stepwise order | Regression |  | Analysis of variance (ANOVA) | | |
| --- | --- | --- | --- | --- | --- |
|  | Coefficients | R^2†^ | d. f. | F | Significance |
| Constant | 25.586 |  |  |  |  |
| Factor 1^‡^ | 12.078 | 0.343 | 1, 27 | 14.069 | <0.002 |
| Factor 3^‡^ | 11.158 | 0.635 | 2, 26 | 22.614 | <0.001 |
| Factor 2^‡^ | 7.704 | 0.774 | 3, 25 | 28.597 | <0.001 |
| ^†^ Step by step cumulative R^2^. | | | | | |
| ^‡^ Factor Score 1, Factor Score 3 and Factor Score 2 correspond to Principal components 1, 3 and 2 in Table 6. | | | | | |
